# Resting-state EEG measures cognitive impairment in Parkinson’s disease

**DOI:** 10.21203/rs.3.rs-2666578/v1

**Published:** 2023-03-20

**Authors:** Md Fahim Anjum, Arturo Espinoza, Rachel Cole, Arun Singh, Patrick May, Ergun Uc, Soura Dasgupta, Nandakumar Narayanan

**Affiliations:** University of California San Francisco; University of Iowa; University of Iowa; University of South Dakota; The University of Iowa; University of Iowa; The University of Iowa; University of Iowa

## Abstract

**Background:**

Cognitive dysfunction is common in Parkinson’s disease (PD) and is diagnosed by complex, time-consuming psychometric tests which are affected by language and education, subject to learning effects, and not suitable for continuous monitoring of cognition.

**Objectives:**

We developed and evaluated an EEG-based biomarker to index cognitive functions in PD from a few minutes of resting-state EEG.

**Methods:**

We hypothesized that synchronous changes in EEG across the power spectrum can measure cognition. We optimized a data-driven algorithm to efficiently capture these changes and index cognitive function in 100 PD and 49 control participants. We compared our EEG-based cognitive index with the Montreal cognitive assessment (MoCA) and cognitive tests across different domains from the National Institutes of Health (NIH) Toolbox using cross-validation schemes, regression models, and randomization tests.

**Results:**

We observed cognition-related changes in EEG activities over multiple spectral rhythms. Utilizing only 8 best-performing EEG electrodes, our proposed index strongly correlated with cognition (rho = 0.68, *p* value < 0.001 with MoCA; rho ≥ 0.56, *p* value < 0.001 with cognitive tests from the NIH Toolbox) outperforming traditional spectral markers (rho = −0.30 – 0.37). The index showed a strong fit in regression models (R^2^ = 0.46) with MoCA, yielded 80% accuracy in detecting cognitive impairment, and was effective in both PD and control participants.

**Conclusions:**

Our approach is computationally efficient for real-time indexing of cognition across domains, implementable even in hardware with limited computing capabilities, making it potentially compatible with dynamic therapies such as closed-loop neurostimulation, and will inform next-generation neurophysiological biomarkers for monitoring cognition in PD and other neurological diseases.

## Introduction

Cognitive dysfunction is a major non-motor symptom of Parkinson’s disease (PD), affecting ~ 20% of the individuals with PD at initial diagnosis and leading to dementia in more than 80% of the individuals with disease progression and aging^1–4^. Because PD-related cognitive symptoms can predict morbidity and mortality, early diagnosis may help counsel families and guide crucial treatment decisions. Detailed neuropsychological testing^5^ is the gold standard for determining cognitive function. However, neuropsychological testing requires several hours and trained examiners and is subject to learning effects, especially with frequent testing^5,6^. Therefore, it is not readily compatible with repeated measurements throughout the day or even within short time intervals such as days or weeks. These features limit its role in capturing cognitive fluctuations in PD or real-time feedback for neuromodulation therapies where cognition can be negatively affected while improving motor function^7–9^.

Short screening tests such as Montreal Cognitive Assessment (MoCA)^10^ or computerized batteries such as National Institutes of Health Toolbox Cognitive Battery^11^ (NIH-Toolbox) have similar limitations. MoCA is a high-performing screening tool for detecting PD-related cognitive impairment^12–15^. Computer-based cognitive tests provided by the NIH-Toolbox are also commonly used as standardized measures of cognition for insights into different cognitive domains^16^. While these cognitive measures can be readily measured, they depend on paper or digital text input formats limited by the patient’s motor and language proficiency, as well as by trained examiners during data collection and scoring^17^.

EEG provides a widely available, low-cost measure of cortical neurophysiology which can provide predictive electrophysiological markers for monitoring instantaneous and long-term cognitive functioning^18,19^. EEG-based indexing of PD-related cognitive functions can generate objective, rapid, and robust measures of cognition with continuous measurements in complex environments. More importantly, it can contribute to easily repeatable longitudinal assessments of cognition with disease progression, provide a screening process for cognitive profiling of the patients, and refine PD treatments such as closed-loop neurostimulation for avoiding cognitive side effects and improving cognitive symptoms^9,20^. In previous studies, EEG-based analysis has facilitated a more accurate characterization of granular fluctuations and it is sensitive to the abnormalities in cortical function that precedes the occurrence of overt disease manifestations^21,22^. EEG recording procedure is generally less stressful and well-tolerated by individuals with cognitive impairments. In addition, EEG-based cognitive measures are more objective and reproducible compared to neuropsychological testing due to the inherent variations in test procedures and inter-rater variabilities^21^. Thus, cognitive assessment by readily available EEG that does not require expert administration and is compatible with repeated or continuous measurement would be of great benefit. Changes in EEG activities correlate with cognitive measures such as MoCA and cognitive tests from NIH-Toolbox in various medical conditions^18,19,23^. However, to date, few EEG-based biomarkers have been found in PD patients that correlate strongly with cognitive performances measured by these tests even though such EEG-based measures can achieve superior assessment of cognitive fluctuations^21,23,24^.

While changes in EEG over multiple spectral rhythms, power ratios, and phase-couplings can correlate with cognition and achieve moderate correlation with several psychometric tests^22,25–29^, most prior EEG-based approaches have focused on specific spectral ranges leading to a limited capturing of EEG activities related to cognition. We recently developed a novel data-driven approach termed Linear predictive coding EEG Algorithm for Parkinson’s Disease (LEAPD) that can efficiently capture spectral EEG profiles over a broad frequency range and reliably detect PD and PD-related depression^7,30,31^. In this study, we hypothesized that cognition-related changes in EEG activities occur in multiple spectral rhythms across the power spectrum in PD and efficient capturing of these changes can index cognitive functions. We utilized LEAPD for capturing these EEG spectral changes related to cognition in a holistic fashion and developed an EEG-based biomarker to index cognition in PD. Our goal was to develop a cognitive assessment tool based on the neurophysiological changes in cortical activities as observed by EEG which would be quick, independent of any specific language and trained expertise while leading to a better understanding of the neurophysiology of cognitive impairment in PD.

In this study, we collected resting-state EEG from 100 individuals with PD and 49 control participants, conducted traditional spectral analyses, and optimized LEAPD to estimate general cognitive function as measured by the MoCA and domain-specific cognitive function using tests from NIH-Toolbox. Our spectral analyses revealed synchronous changes related to cognitive measures over a broad spectrum in EEG. We found that LEAPD can efficiently capture these changes, strongly correlates with MoCA scores as well as with cognitive scores from NIH-Toolbox, and can detect MoCA-defined cognitive impairment in PD and control participants. These results not only provided evidence supporting our hypothesis on cognition-related changes in EEG activities but also delivered cognitive measures based on changes in cortical activities. Specifically, strong correlations with MoCA and other well-established cognitive measures indicate the effectiveness of EEG to quickly gauge cognitive impairment that can lead to EEG-based diagnostic technologies for detecting cognitive outcomes quickly and accurately, real-time feedback on the cognitive effects of medical and surgical interventions, and long-term monitoring of cognitive function in PD and other neurodegenerative diseases.

## Methods

### Participants and cognitive measures

We recruited 100 PD participants from the Movement Disorders Clinic at the University of Iowa, Iowa City between 2017 and 2022 (Table 1). A movement disorders physician diagnosed each individual with PD according to the United Kingdom Brain Bank criteria. All procedures were performed while participants were taking their usual medications. Additionally, 49 demographically similar controls without known neurological disease were recruited from the general Iowa City community between 2017 and 2022 through the Seniors Together in Aging Research registry (https://icts.uiowa.edu/star). The motor component of the United Parkinson’s Disease Rating Scale and the MoCA scores were administered by trained raters. Participants also completed other clinical assessments of cognition, mood, and gait. The study was approved by the University of Iowa Institutional Review Board (protocol # 201707828). Written informed consent was provided by all participants. We used MoCA to quantify cognitive condition among participants as it is more sensitive to cognitive deterioration in PD ^12–14^. We defined cognitive impairment as MoCA scores < 26 and cognitively normal as MoCA scores 26–30^10,15,32,33^. Among all 149 participants, 53 PD and 11 control participants had MoCA-defined cognitive impairment (MoCA score < 26). The control participants with cognitive impairment had no pre-existing neurological or psychiatric disease. The median MoCA score among all participants was 26. To assess specific aspects of cognitive functioning, we used NIH-Toolbox, a standardized computer-based neuropsychological screening battery^11^. Five tests from the NIH-Toolbox were administered to the participants: Picture vocabulary test (PVT), Pattern comparison processing speed test (PCPST), Dimensional change card sorting test (DCCST), Flanker inhibitory control and attention test (FICAT), and Picture sequence memory test (PSMT). The tests were administered through iPads and extensive user directions were provided by the examiner.

The goal of this study was to investigate LEAPD as an index for PD-related cognitive impairment. To modify and optimize LEAPD for MoCA, EEG recordings from the 149 participants were divided into two groups: cognitively impaired participants (MoCA score < 26; 64 total participants; 53 PD, 11 control) and cognitively normal participants (MoCA score ≥ 26; 85 total participants; 47 PD; 38 control; Table 1). To optimize training models for each of the cognitive tests from NIH-Toolbox, all participants were similarly divided into two respective training groups based on their scores (median as cut-off) while the classification of normal cognition and cognitive impairment was based on only MoCA scores.

### EEG recordings

**Figure 1:**
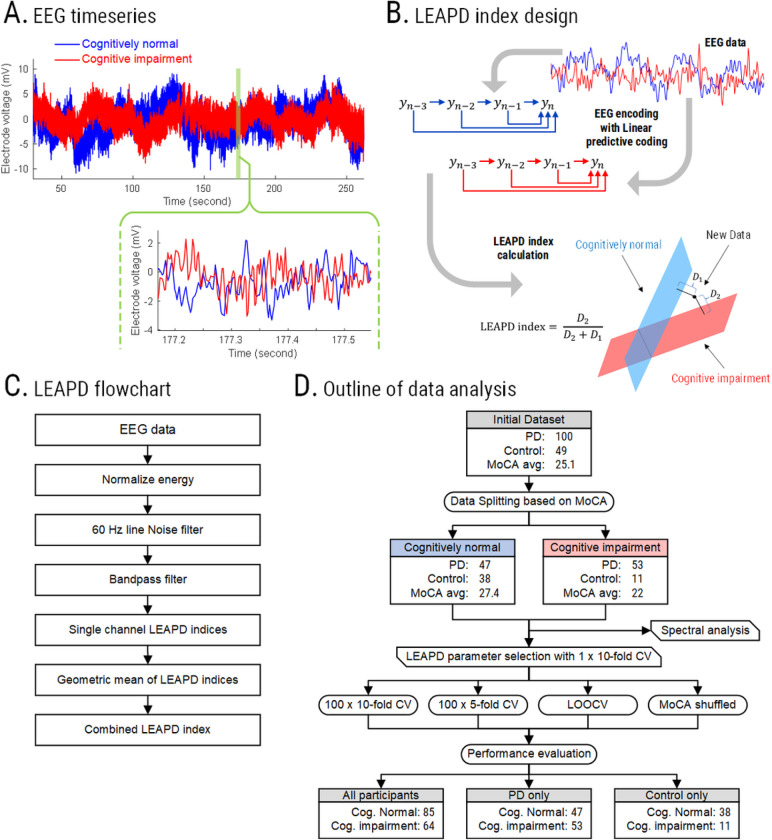
Methodology of the study. (**A**) EEG time series comparison between a Parkinson’s disease participant with normal cognition (*blue*) and a participant with cognitive impairment (*red*) from a representative electrode P8. (**B**) Illustration of single-electrode LEAPD index generation using separate affine subspaces for cognitively impaired (*red*) and cognitively normal (*blue*) participants in the feature space.D1 and D2 are the distances from the affine subspace of the cognitively normal (*blue*) and cognitively impaired participants (*red*), respectively. (**C**) Steps for LEAPD index generation from EEG data. (**D**) Schematic and data analysis outline of the study for LEAPD and traditional EEG spectral analysis with randomization test and cross-validations for MoCA. Abbreviation: PD = Parkinson’s disease. LOOCV = Leave-one-out cross-validation. MoCA = Montreal Cognitive Assessment.

Resting-state EEG was collected from participants while they sat in a quiet room with their eyes open for a few minutes ON dopaminergic medications as usual (Fig. 1A) within 1 hour of MoCA administration using a 64-channel actiCAP and Brain Vision system (Brain Products GmbH) with a 0.1-Hz high pass filter and a sampling frequency of 500 Hz. Data from 60 electrodes were used for each participant (Pz was the online reference and Iz, I1, and I2 were inconsistent among the participants and were excluded). EEG data from each electrode were normalized to unit power, and 60-Hz line noise was removed. No further manual inspection, cleaning, or artifact removal was conducted.

### LEAPD: EEG-based feature for cognition

LEAPD is a data-driven approach originally developed as an efficient EEG-based marker for PD that can reliably detect PD-related changes in preclinical animal models as well as in humans while strongly outperforming prior methods and can identify clinical depression in PD^7,30,31^. The key aspect of LEAPD is the use of linear predictive coding (LPC) for encoding EEG time series and efficiently capturing its unique spectral profile by capturing dominant oscillatory modes and encoding them into a few LPC parameters^30,31,34^. In this study, we utilized LEAPD to capture cognitive function by encoding EEG data using LPC and finding separate affine subspaces for cognitively impaired and cognitively normal participants. We hypothesized that after encoding EEG into vectors of LPC coefficients, there are two distinct affine subspaces (i.e., subspace for cognitive impairment which corresponds to low cognitive score, and subspace for normal cognition which corresponds to high cognitive score) such that the relative distances from the two affine subspaces (LEAPD indices) highly correlate with cognition and can detect cognitive impairment. (Fig. 1B). Single channel LEAPD indices were calculated from individual electrodes and their geometric means provided a combined LEAPD index (Fig. 1C, Appendix A1). After optimizing LEAPD parameters, we applied several cross-validation schemes and performed randomization tests by randomly shuffling cognitive scores among participants to evaluate performance (Fig. 1D, Appendix A2).

### Statistical analysis

As the distribution of the bounded LEAPD index is non-gaussian, we utilized the Spearman (rank) partial correlation method controlling for the participants’ age to measure correlation while accounting for group-level age differences (Table 1) ^35,36^. All statistical procedures (details in Appendix A3) were performed using MATLAB 2021b and R (version 4.1.2). All data and statistical analyses were reviewed by the Biostatistics and Research Design Core in the Institute for Clinical and Translational Science at the University of Iowa.

## Results

### Correlation of EEG spectral features and LEAPD with cognitive measures

**Figure 2:**
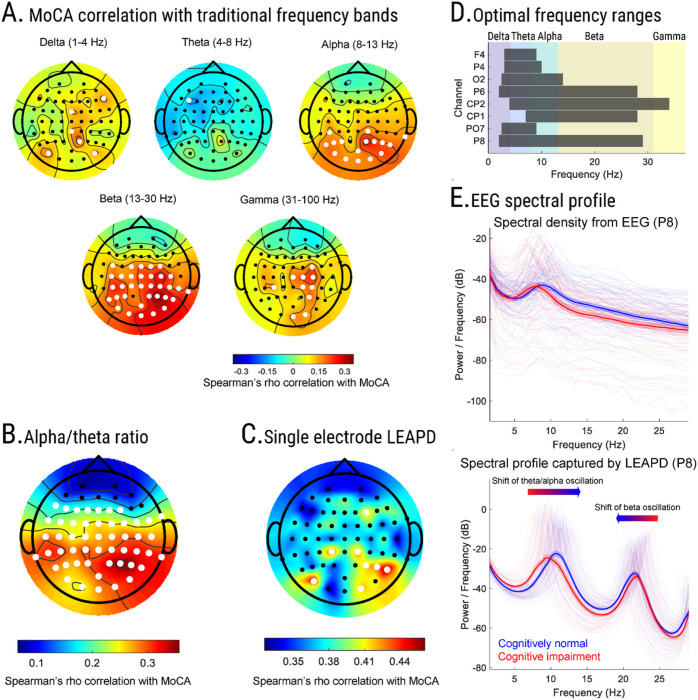
EEG feature analysis and parameter choice. Topographic plots of age-adjusted Spearman’s rho correlation between MoCA scores and (**A**) traditional frequency bands, (**B**) Log-spectral ratio of Alpha (8 – 13 Hz) and Theta (4 – 8 Hz) rhythm. Electrodes marked as white signifies a statistically significant correlation (*p*value < 0.05). (**C**) Topographic plot of correlations between single-electrode LEAPD indices and MoCA scores during optimal parameter selection for combined LEAPD index. Selected electrodes are marked as white.(**D**) Optimal frequency ranges for the selected EEG electrodes. Vertical color blocks represent traditional frequency bands. (**E**) Comparison of spectral densities between cognitively impaired (red) and cognitively normal (blue) groups from single-electrode (*P8*) raw EEG data (*top*) and EEG-encoded linear predictive coding models (*bottom*) capturing unique spectral profiles. Thick lines show mean spectral densities; lighter lines are individual spectral densities, and shaded areas show standard error of the mean. The arrows mark the directions of the shifts of the spectral peaks from cognitive impairment to normal cognition. Data in panel A, B, C, and E are from all participants (n=149).

First, we correlated LEAPD and traditional spectral EEG features with MoCA score, a screening tool for cognition^5,33^. Spectral analyses of EEG revealed moderate correlations with MoCA scores (Fig. 2A–B). In particular, MoCA score showed statistically significant correlations with increased beta (13 – 30 Hz) power in central-parietal region (highest at P4: rho = 0.37, *p* value < 0.001), increased alpha (8 – 13 Hz) power in parietal region (highest at P2: rho = 0.29, *p* value < 0.001), increased gamma (31 – 100 Hz) power in central-parietal region (highest at C4: rho = 0.25, *p* value = 0.002), increased delta (1 – 4 Hz) power in central-parietal region (highest at P2: rho = 0.25, *p* value = 0.002) and reduced theta (4 – 8 Hz) power in left frontal region (F5: rho = −0.17, *p* value = 0.04). For investigating the correlation between spectral ratios and MoCA scores, we chose the alpha/theta log-spectral ratio^29^ which simultaneously captures reduced theta and increased alpha power and showed a statistically significant correlation in almost all regions with MoCA (highest at P4: rho = 0.36 *p* value < 0.001, Fig. 2B). Furthermore, the highest correlation in beta power and alpha\theta ratio were both in the parietal region (P4) implicating the possibility of achieving high correlations with holistic approaches that can capture changes in EEG activities over a broad spectrum.

Next, we tested the hypothesis that LEAPD, which captures the spectral profile of EEG over a broad range, can measure cognitive function in PD. Individual electrode performances demonstrated that LEAPD indices from single electrodes strongly correlated with MoCA score (Fig. 2C; Spearman’s rho > 0.41 noted for P8, PO7, CP1, CP2, P6, O2, P4, and F4). We selected these eight electrodes to calculate a combined LEAPD index. Optimal LEAPD frequency ranges for the central-parietal electrodes (P6, CP1, CP2, and P8) were broad while PO7, O2, P4, and F4 were focused on lower frequencies (Fig. 2D). Differences in EEG spectral densities on a broad spectrum were apparent in the data-driven central-parietal electrodes between cognitively impaired and cognitively normal participants, regardless of whether they had PD which was consistent with our spectral power analyses (Fig. 2E). These differences were captured by the EEG spectral profiles of LPC models through the shift of alpha/theta oscillation and beta oscillation (Fig. 2E). Correlations between MoCA score and the combined LEAPD index were remarkably high (rho = 0.68, *p* value < 0.001) across all cross-validation schemes (±0.016; Table 2; Supplementary Table S1). MoCA score correlations with LEAPD indices were two times higher and significantly stronger than those from spectral analyses (LEAPD vs. beta power from P4: z = −4.4, *p* value = < 0.001; rho = 0.7 vs. 0.37). MOCA and LEAPD index correlations were also twice as high as the highest previously reported MoCA correlation achieved by EEG features in PD^24^. Correlations with MoCA scores were similar (rho = 0.66 ± 0.024) for participants with PD alone, but lower in control participants (rho = 0.46 ± 0.006; Table 2; Supplementary Table S1). During the randomization test where we shuffled MoCA scores among all participants, MoCA correlations obtained by LEAPD were very low and not statistically significant (rho=0.02, *p* value = 0.77; Supplementary Table S2).

We also correlated LEAPD and EEG spectral powers with cognitive scores from the NIH-Toolbox (Supplementary Fig. S3). Alpha and beta power correlated with PVT scores in the parietal region with the highest correlation of 0.24 (beta power at CP3, *p* value <0.005). PCPST, PSMT and DCCST scores correlated with decreased theta and increased beta power with the highest correlation of −0.29 (theta power at C6, *p* value <0.001), −0.30 (theta power at T8, *p* value <0.001), and 0.23 (beta power at PO8, *p* value <0.001) respectively. Alpha, beta and gamma power showed significant correlations with FICAT scores with the highest correlation of 0.36 (beta power at P4, *p* value <0.001). In contrast, the combined LEAPD index showed high correlations with the cognitive scores across all cross-validations (rho in PVT: 0.64 ± 0.01, PCPST: 0.58 ± 0.004, DCCST: 0.68 ± 0.02, FICAT: 0.61 ± 0.01, PSMT: 0.56 ± 0.007; Table 2, Supplementary Table S1, Supplementary Fig. S4). No statistically significant correlation was found between LEAPD and NIH-Toolbox cognitive scores in the randomization test for all participants (Supplementary Table S2). While we presented correlation results using Spearman’s rho, we observed similar results with Pearson correlation. Taken together, these data demonstrate that LEAPD index could strongly correlate with cognition in PD.

### Detection of cognitive impairment

**Figure 3:**
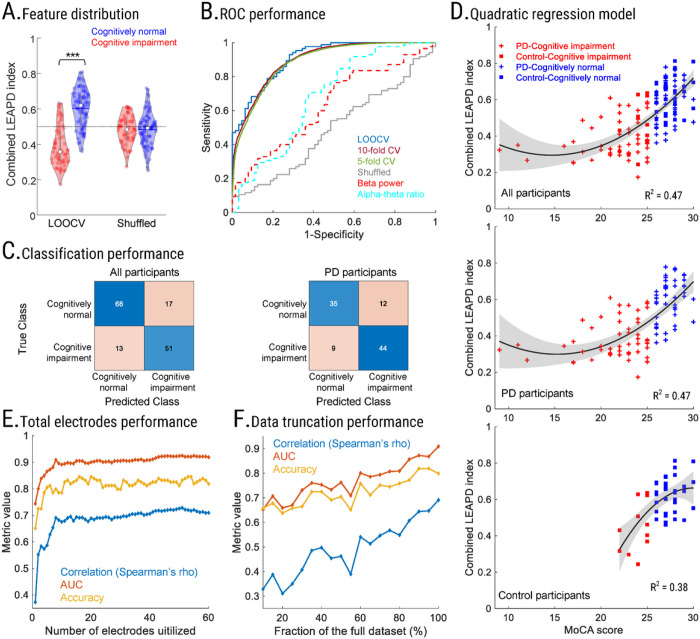
Performance evaluation of LEAPD. (**A**) Violin plots of LEAPD indices for cognitively impaired (*red*) and cognitively normal (*blue*) participants during LOOCV before (*left*) and after MoCA score shuffling among participants (*right*). (**B**) Receiver operative characteristic (ROC) curve for LEAPD in various cross-validations as well as in shuffled MoCA data (solid lines), and for the top-performing traditional spectral features (dashed lines; beta power and alpha-theta log ratio at P4). (**C**) Classification performances of LEAPD with confusion matrices for all participants (*left*) and Parkinson’s disease only (*right*) during LOOCV. (**D**) Scatter plot for the quadratic regression model between MoCA and LEAPD indices for all participants (*top*; n=149), Parkinson’s disease-only (*middle*; n=100), and controls (*bottom*; n=49) during LOOCV. (**E**) Spearman’s rho correlation with MoCA (*blue*), classifier accuracy (*yellow*), and AUC (*red*) performance of LEAPD while varying the number of EEG electrodes utilized for the combined LEAPD index. The x-axis is the number of EEG electrodes utilized, and the y-axis is the metric (AUC, classifier accuracy, or Spearman’s rho value). (**F**) Robustness of LEAPD performance in terms of correlation with MoCA (*blue*), classifier accuracy (*yellow*), and AUC (*red*) while truncating the dataset. The x-axis is the size of the dataset after truncation compared to the original size in percentage. Results in panel A, B, E, and F are from all participants (n=149). Abbreviation: PD = Parkinson’s disease, Leave-one-out cross-validation = LOOCV, Cross-validation = CV.

For all participants, LEAPD classified cognitive impairment and normal cognition with 0.90 ± 0.01 AUC (area under the receiver operating curve), 80.44 ± 0.55% classifier accuracy, 79.52 ± 0.65% sensitivity, and 81.14 ± 0.99% specificity across all cross-validation schemes (Fig. 3A–C; Table 2; Supplementary Table S1; rank-sum test on LEAPD scores for cognitive impairment vs. cognitively normal group: *p* value < 0.001) while the top performing traditional spectral features yielded moderate AUC (alpha-theta ratio: 0.68; beta power: 0.63; electrode P4; Fig 3B). In PD participants only, LEAPD performance was similar to the case of all participants (0.90 ± 0.01 AUC, 78.90 ± 0.09% classifier accuracy, 82.62 ± 0.55% sensitivity, and 74.70 ± 0.58% specificity). This classification of PD-related cognitive impairment was stronger than previous studies (AUC was 17.6% higher)^37^. For control participants only, LEAPD demonstrated higher classifier accuracy and specificity (83.59 ± 1.87% classifier accuracy, 89.11 ± 2.03% specificity), but lower AUC and sensitivity (0.85 ± 0.02 AUC, 64.55 ± 1.82% sensitivity). In the randomization test with MOCA-shuffled data, the results were close to chance (50.34% classifier accuracy, 0.49 AUC, *p* value = 0.86; Supplementary Table S2). Finally, we checked the potential effects of the group sizes by subsampling the dataset with equal numbers of cognitively impaired and cognitively normal participants in leave-one-out cross-validation which provided similar performances (0.91 ± 0.02 AUC, 79.70 ± 2.79% classifier accuracy, 79.60 ± 3.50% sensitivity, and 79.80 ± 2.90% specificity; Supplementary Figure S6) to the full dataset (Table 2).

### Linear vs. quadratic relationships

We noted that the correlation between the combined LEAPD indices and MoCA scores was unlikely to be linear. To explore this relationship, we used regression analyses. Both linear and quadratic regression models for the combined LEAPD index and MoCA scores were statistically significant (linear and quadratic *p* values < 0.001; Fig. 3D; Table 2; Supplementary Table S1). However, we found that R^2^ increased by 7% from linear to quadratic regression (quadratic: 0.46 ± 0.018; linear: 0.39 ± 0.016; Table 2, Supplementary Table S1) across all cross-validations, and the quadratic model had a statistically significant better fit compared to the linear model (likelihood ratio test with leave-one-out cross-validation; *p* value < 0.001). Regression models for MoCA-shuffled data were not statistically significant (Supplementary Table S2).

Quadratic regression models achieved higher R^2^ for PD participants compared to control participants (Table 2). No statistically significant effect of PD was observed after including it in the quadratic model as a random effect (mixed-effect vs. no-effect: *p* value = 0.98; Fig. 3D). The same conclusion was obtained for linear regression (mixed-effect vs. no-effect: *p* value = 0.35). No additional assumptions were made during the regression analyses. These data suggest a quadratic relationship between the combined LEAPD index and MoCA scores that is statistically independent of PD, and that the algorithm might be triggered by aspects of the EEG signals related to cognitive status, irrespective of PD.

### LEAPD robustness

To investigate the robustness of LEAPD, we examined how correlation and classification performances vary with the number of electrodes (1–60; Supplementary Video S5). The correlation of the combined LEAPD index with MoCA score and the AUC of cognitive classification both reached their respective maximums with 51 electrodes while the maximum classifier accuracy was achieved with 16 electrodes (Fig. 3E). However, with more than 10 electrodes, improvements in correlation, AUC, and classifier accuracy were marginal. During data truncation with 10% of the data, LEAPD achieved an AUC of 0.65 (Avg. EEG length = 16 seconds; rho = 0.33, 65.77% classifier accuracy; Fig. 3F). With 40% of the data, it achieved 0.75 AUC (Avg. EEG length = 1 minute; rho = 0.5, and 72.48% classifier accuracy). The results during data truncation show a gradual increase in performance indicating that the algorithm might benefit from longer EEG recordings. Performances of LEAPD in MoCA-shuffled data (randomization test) during data truncation were not statistically significant (45.57 ± 3.22% classifier accuracy, 0.44 ± 0.03 AUC, rho = −0.07 ± 0.06). Taken together, these results indicate that LEAPD can powerfully identify PD-related cognitive impairment from a limited electrode montage and a few minutes of resting-state EEG.

## Discussion

In this study, we hypothesized that EEG activities change in a wide frequency range across the power spectrum with cognition and capturing these changes can lead to EEG-based cognitive measures. Our spectral analyses indicated that EEG activities over multiple spectral rhythms correlate with cognitive measures. We found that LEAPD, a data-driven machine learning algorithm^31^, can efficiently capture EEG spectral profiles for cognition and detect cognitive dysfunction both in PD and control participants. Our data showed that by rapidly encoding resting-state EEG data from eight electrodes, LEAPD can accurately index cognitive function as measured by MoCA or by the cognitive tests from the NIH toolbox. It also detected cognitive impairment in control participants, performed consistently across multiple cross-validation schemes, provided reliable performance during data truncations, and showed no indication of overfitting in the randomization tests. The lack of significant correlation between LEAPD and cognitive tests during training and validation using data with randomly-shuffled cognitive scores showed the dependency of LEAPD’s performance on capturing the neurophysiological changes related to cognition. Thus, LEAPD can be useful for finding novel EEG-based biomarkers for cognitive impairment in humans including individuals with PD.

Our analyses showed both a linear and a quadratic relationship between LEAPD indices and MoCA scores. However, the quadratic relationship was stronger, suggesting a complex relationship between cortical neurophysiology and cognition. The bounded non-gaussian feature of LEAPD scores, combined with this predominantly nonlinear, albeit monotonic, relationship with MoCA scores, made Spearman’s rho a more robust measure of correlations compared to Pearson’s^35,36^.

Strong correlations of LEAPD-based cognitive indices with PVT, DCCST, PCPST, PSMT, and FICAT scores in addition to MoCA suggest that cognitive impairment involves dysfunction in cortical networks that manifest in scalp EEG^21,38^ and that our approach is sensitive to cognitive function across different domains. Although various other measures have been used to assess cognitive impairment via EEG^38–40^, to the best of our knowledge no other technique has this level of accuracy and correlation in PD patients and is as computationally-efficient. While LEAPD was useful in both PD and demographically similar control participants, its lower performance with control participants might be due to the limited range of performance in this healthy group with a small number of cognitively impaired participants. At its core, LEAPD is a data-driven method originally designed for PD vs control classification that captures characteristic spectral changes and can differentiate between data population groups such as PD and control groups or PD with and without depression with high accuracy^7,31^. In this study, LEAPD was able to provide cognitive markers in both PD and control participants by rapidly capturing the EEG spectral profiles while showing no significant effects of PD in the detection of cognitive impairment. This general effectiveness of LEAPD indices might lead to early diagnosis of cognitive impairment in not only PD but also other brain diseases that impair cognition that manifests through EEG such as Dementia with Lewy bodies (DLB)^21^.

Although purely data-driven, the optimal frequency bands revealed by LEAPD showed neurophysiological significance and correlation with cognitive measurements in previous studies. For example, the majority of optimal LEAPD spectral bands for MoCA correlation include theta and delta rhythms which are predictive of MoCA scores, have strong correlations with several cognitive measures, and reflect high-level cognitive processes and mechanisms for cognitive control^24,25,28,41^. Furthermore, changes in EEG activities related to cognitive function can occur synchronously over multiple spectral rhythms. For example, delta-alpha coupling correlates with cognitive measures^25^, and cue-related medial frontal delta and theta power correlate with cognitive function in PD^24,42^. Abnormal delta and alpha rhythms in the posterior brain regions can index the decline of cognitive visuo-spatial function^38^. Delta, theta, alpha, and beta activities in EEG and their ratios reflect cognitive function in PD^22,37,42,43^. In our study, MoCA correlations of alpha-theta ratio and beta power in specific regions indicated synchronous changes in EEG over a broad spectrum. While changes in multiple EEG rhythms are traditionally captured with spectral ratios, which can correlate with cognitive measures in PD^26,27,29^, these ratios are limited by specific choices of spectral rhythms. In contrast, LEAPD is not strictly confined to the traditional spectral rhythms and captures simultaneous changes in multiple EEG rhythms by extracting the spectral profile in a holistic fashion, which led to an efficient estimation of cognitive dysfunction in this study. In line with previous studies, our results also indicated that the neurophysiological changes related to cognitive fluctuation are encoded mostly in spectral features rather than intra-channel connectivity measures^20^. Furthermore, the LPC models in LEAPD can reveal changes in the underlying oscillatory modes of the EEG signal. In our study, the dominant theta-alpha and beta oscillations captured by LEAPD at the optimal central-parietal electrodes indicated gradual shifts with cognitive function. Interestingly, spectral peak shifts of theta-alpha EEG rhythms in posterior cortical regions can predict dementia development in PD^39,44^. Shifts of peak frequency from alpha to theta with cognition, as discovered by LEAPD (Fig 2E), have also been observed previously in PD^20^. Future studies will investigate the oscillatory modes captured by LEAPD for a better understanding of the underlying neurophysiological mechanisms. Finally, unlike prior studies^23^, we analyzed resting-state EEG activities that were not influenced by the cognitive measurement procedures.

EEG-based cognitive marker provided by LEAPD might be a useful complement to classical neuropsychological tests by choosing candidates as a large-scale screening and follow-up tool for further detailed testing leading to a more feasible utilization of such valuable resources and trained health professionals that are sometimes not immediately available^45^. Because LEAPD uses LPC to compress key features of EEG spectra, it is computationally efficient and suitable for real-time applications^30,31^. In our study, LEAPD required at most eight electrodes eliminating the need for high-density scalp-EEG and can be implemented in wearable EEG devices^46^. It can capture EEG-based cognitive markers within a few minutes (Table 1) and thus could effectively identify the characteristic cognitive fluctuations of DLB or PD dementia^21,39^. Complementary strengths of LEAPD to neuropsychological testing include speed, repeatability, and independence from patient-related factors such as fatigue and inability to participate due to severe motor symptoms or dementia. Future longitudinal studies are needed to explore these aspects. Furthermore, combined with advanced therapies such as adaptive neurostimulation with long-term wireless streaming of neural recordings^47^, EEG-based assessment of cognitive function by LEAPD might help design better feedback signals or select appropriate neurostimulation settings in targeted treatments for mitigating stimulation-related cognitive side effects and identify patients at risk in future studies^9^.

Our study has several limitations. First, scalp EEG has poor spatial resolution whereas MEG, fMRI, or intraoperative recording studies might provide detailed spatial information about brain activity, which could contribute to more accurate cognitive measurements of LEAPD. Despite the poor spatial resolution, we chose EEG as it is widely available, low-cost, and has shown promising results as a neurophysiological marker compared to others including fMRI, SPECT, PET, and neurophysiological tests in neurological conditions such as DLB^21^. Second, MoCA-based classification (cut-off 26/30) for cognitive impairment may not be as accurate as classification using Level II testing for cognitive impairment in PD^5^. However, our primary goal of this study was to index cognition using EEG that strongly correlates with standard measures of cognition. Third, our study is cross-sectional and represents a moderately sized convenience sample. Longitudinal studies with larger cohorts with repeated measurements are needed to determine the predictive value of our EEG-based markers on cognitive outcomes and their usefulness in monitoring cognition in PD in clinical practice. Fourth, our study was ON levodopa as usual, although our past work indicates that levodopa does not change EEG spectra, and our previous work showing levodopa differences was in rodent striatal field potentials, which are vastly different than scalp EEG^30,48^. Lastly, we had a moderate-sized dataset and thus evaluated the performance of LEAPD scores using cross-validation schemes, commonly utilized in measuring diagnostic performances^49,50^. Furthermore, we implemented subject-size cross-validation technique, the proper way of estimating a model’s performance in a diagnostic scenario^49,50^ with various values in k-folds, and repeated the process 100 times with independent shuffling of the folds. Performance variations of LEAPD indices across the cross-validation schemes were low (≤ 2% in correlations with cognitive measures and detection of cognitive impairment; Table 2; Supplementary Table S1) and robustness analyses showed stable performance. In addition, we trained LEAPD on data with randomly shuffled cognitive scores among subjects which showed no statistically significant correlation with cognitive scores or detection of cognitive impairment suggesting that the correlations with cognitive measures are not achieved through fitting noise or data artifacts.

In summary, we found that LEAPD can provide a data-driven feature from resting-state EEG for cognitive measures in PD. This could be useful as a potential marker to infer cognitive impairment in PD or other neurological diseases. Because LEAPD is scalable and amenable to real-time applications, it also might inspire novel feedback-based interventions or advanced neuromodulation therapies targeted at cognition.

## Data Availability

The datasets and the underlying codes generated and/or analyzed during the current study will be available in a public repository upon publication at: http://narayanan.lab.uiowa.edu.

## Abbreviations

PD: Parkinson’s disease
AUC: Area Under the receiver operating characteristic Curve
LEAPD: Linear predictive coding EEG Algorithm for Parkinson’s Disease
LPC: Linear Predictive Coding
MoCA: Montreal Cognitive Assessment
NIH: National Institutes of Health
PVT: Picture vocabulary test
FICAT: Flanker inhibitory control and attention test
DCCST: Dimensional change card sorting test
PCPST: Pattern comparison processing speed test
PSMT: Picture sequence memory test
